# A Decision Tree Model for Analysis and Judgment of Lower Limb Movement Comfort Level

**DOI:** 10.3390/ijerph19116437

**Published:** 2022-05-25

**Authors:** Zhao Xu, Weijie Pan, Yukang Hou, Kailun He, Jian Lv

**Affiliations:** Key Laboratory of Advanced Manufacturing Technology of the Ministry of Education, Guizhou University, Guiyang 550025, China; zxu9990@gmail.com (Z.X.); 376190399h@gmail.com (W.P.); gs.ykhou21@gzu.edu.cn (Y.H.); hkl0006000ox@yeah.net (K.H.)

**Keywords:** comfort level, sEMG, biology, decision tree, motion capture

## Abstract

To address the problem of ambiguity and one-sidedness in the evaluation of comprehensive comfort perceptions during lower limb exercise, this paper deconstructs the comfort perception into two dimensions: psychological comfort and physiological comfort. Firstly, we designed a fixed-length weightless lower limb squat exercise test to collect original psychological comfort data and physiological comfort data. The principal component analysis and physiological comfort index algorithm were used to extract the comfort index from the original data. Secondly, comfort degrees for each sample were obtained by performing K-means++ to cluster normalized comfort index. Finally, we established a decision tree model for lower limb comfort level analysis and determination. The results showed that the classification accuracy of the model reached 95.8%, among which the classification accuracy of the four comfort levels reached 95.2%, 97.3%, 92.9%, and 97.8%, respectively. In order to verify the advantages of this paper, the classification results of this paper were compared with the classification results of four supervised classification algorithms: Gaussian Parsimonious Bayes, linear SVM, cosine KNN and traditional CLS decision tree.

## 1. Introduction

The human lower limb is a multi-degree-of-freedom-mechanism composed of complex bones, joints, soft tissues, and skeletal muscles [1], and the lower limb movement is a directional movement which is produced by the skeletal muscles contracting and driving the bones with the joints as fulcrums under the stimulation of the central nerve [1,2]. It has the characteristics of high flexibility, wide range of motion, and strong coordination of various parts in daily life [2]. Persistent poor postural lower limb movements can lead to a certain degree of operational risk and affect operational efficiency [3]. An effective quantitative evaluation method for lower limb motion comfort can reduce the probability of risk during lower limb motion, and avoid occupational injuries caused by prolonged uncomfortable posture [4], it can provide not only quantitative comfort reference and theoretical support for the design and optimization of human–machine performance assisted wear equipment, but also new ideas for monitoring the comfort status in the process of lower limb rehabilitation and medical treatment.

Currently, relevant research institutions in various countries have achieved certain results in human comfort evaluation [5,6,7,8,9,10,11,12,13,14,15,16,17], they are mainly analyses and evaluations of upper limb motion comfort from bioelectrical signal comfort feature extraction [5,6,7,8,9], biomechanical simulation analysis [10,11,12,13,14], and subjective comfort measurement [15,16,17].

Among bioelectrical signal comfort features extraction methods, such as surface electromyography (sEMG) features [5], electroencephalogram (EEG) features [6], etc., sEMG signal is widely used in fatigue detection and comfort monitoring due to its simplicity of operation and non-invasiveness. EMG signal is a one-dimensional voltage time series signal obtained by guiding, amplifying, displaying and recording the bioelectrical signal changes in the neuromuscular system activity by electrodes [7], while sEMG signal is a comprehensive bioelectrical effect with non-linear characteristics formed by superficial muscle EMG and electrical activity on the nerve trunk on the skin surface. sEMG signal is acquired by electrodes on the skin surface for myoelectricity signal, which has non-invasive characteristics [8]. The sEMG signal is collected by electrodes on the skin surface, which is a non-invasive, real-time and multi-target measurement. Matsumoto et al. [9] first proposed a method, electromyogram fatigue threshold (EMG_FT_), to quantify the skeletal muscle load by applying the characteristic values of sEMG signal. The EMG_FT_ algorithm is able to find the threshold point where skeletal muscle enters the anaerobic state, thus evaluating the fatigue state and comfort state of skeletal muscle quantitatively.

In terms of biomechanics and its simulation analysis, rapid upper limb assessment (RULA) [10] and rapid entire body assessment (REBA) [11] are two internationally accepted assessment methods for postural load exposure risk [12]. Studies have shown that both methods have the advantage of short time required, simple methods, lower costs and effectiveness in assessing biomechanical overload [13]. In biomechanical analysis, most researches combine motion capture technology and biomechanics simulation software, such as JACK (Siemens, Berlin, Germany) and Anybody (AnyBody Technology, Aalborg, Denmark), to simulate joint angles, joint moments, limb muscles and loads during limb movement; in this way, the risk, fatigue state and comfort state during limb movement are quantitatively assessed [14].

In the current studies, subjective comfort perceptions are usually measured by subjective scales, such as Likert scales [15], Borg fatigue subjective self-perception scale [16], etc. Subjects need to fill in the questionnaire during the test to reflect the subject’s subjective comfort feelings at that moment, which is a common means of quantifying subjective comfort feelings [17].

**Figure 1:**
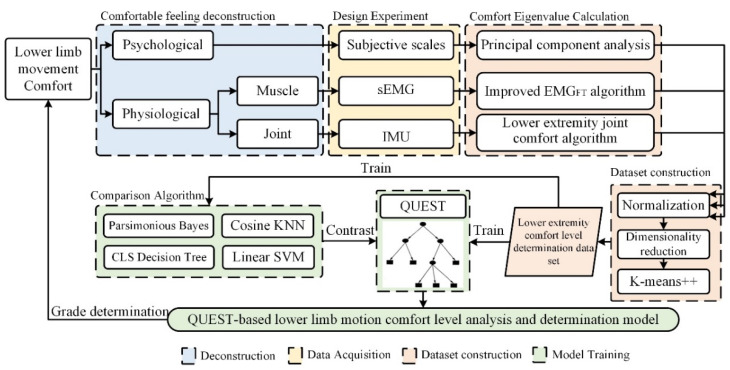
Research method.

However, the matter of comfort feelings of human during lower limb exercise is a complex situation which is influenced by many factors [18]. However, most of the above studies and methods to quantify the comfort feelings during lower limb exercise were only from a single comfort index. To be specific, lower limb exercise involves a large number of collaborative relationships among skeletal muscles, joints and bones. What is more crucial is the degree of physiological comfort largely affects the degree of psychological comfort, and vice versa [19]. Thus, this paper proposed a decision tree model for analysis and judgment of lower limb movement comfort level, which solves the fuzzy and one-sidedness problem of the comprehensive comfort evaluation in the current. Firstly, this paper deconstructs the comfort perception into two dimensions: psychological comfort and physiological comfort. Then, a fixed-length weightless lower limb squat exercise test was designed to collect the subjective scale scores of psychological comfort feelings, original sEMG signals of rectus femoris (RF) and tibialis anterior (TA) muscles and motion capture position information of hip, knee and ankle joints. Secondly, the comfort index was obtained by principal component analysis, the improved EMG_FT_ fatigue level determination algorithm and the lower limb joint angular acceleration variance comfort index algorithm. Then, the comfort degrees for each sample were obtained by performing K-means++ to cluster normalized comfort index. Finally, based on the data set consisting of the comfort index after screening and the comfort degrees, the decision tree model for analysis and judgment of lower limb movement comfort level was established by training. In order to prove the effectiveness and advantages of the model proposed in this paper, the comfort level classification results of this method are compared with four supervised classification algorithms, namely, Gaussian Bayesian [20], linear support vector machine (SVM) [21], cosine k-Nearest Neighbor (KNN) [22] and Concept Learning System (CLS) decision tree [23]. The research method of this paper is shown in Figure 1.

## 2. Materials and Methods

### 2.1. Materials

In order to collect the psychological and physiological data related to the upper limb comfort characteristics, this paper designed a fixed-length weightless lower limb squat exercise test.

In the squatting action, the rectus femoris muscle of the human lower limb starts from the lateral thigh root, below the iliac bone in front of the lower iliac spine and the upper edge of the acetabulum, and wraps around the front of the knee after the patella, ending at the tibial ramus in front of the upper tibia by the patellar ligament, which has the role of extending the knee joint and flexing the thigh. The anterior tibialis is one of the anterior calf muscles, which allows the foot to dorsiflex and turn inwards on the center of the foot, and when the foot bone is fixed, it contracts with the other muscles to bring the lower leg forward. The hip, knee and ankle joints cooperate with each other to complete the contraction during the squatting movement of the lower limb, and therefore the RF and TA are chosen as the skeletal muscles and the hip, knee and ankle joints as the joints.

So, we simultaneously collected the subjective psychological comfort scale scores, sEMG signals of the right side RF and TA [24] muscles of the right lower limb, as well as the motion capture position information of the hip, knee and ankle joints during the lower limb squat exercise.

#### 2.1.1. Test Equipment

In order to collect the sEMG signal, lower extremity joint position information and subjective perception of comfort during the test, the TrignoTM Wireless EMG acquisition system from Delsys, MA, USA, was used to collect the sEMG signal at 1925 Hz and the motion capture signal at 75 Hz. The subjective comfort level was measured on a 7-point Likert scale. The subjective comfort Likert scale consisted of four questions, Q1: subjective rating of lower limb comfort, Q2: subjective rating of lumbar comfort, Q3: subjective rating of respiratory rhythm level, and Q4: subjective rating of skeletal muscle fatigue. The subjective perception of comfort Likert 7 scale was filled out by the tester by asking the subject during the test, and the tester performed the questioning every 10 s during the test.

#### 2.1.2. Subjects

Twenty-five healthy male volunteers, aged (22.4 ± 1.5) years, height (173.2 ± 5.8) cm, weight (65.3 ± 7.2) kg, and body mass index (BMI) of 22.7 ± 1.8, were selected for this trial. All volunteers were students of Guizhou University, and the data were collected at the Key Laboratory of Modern Manufacturing Technology of the Ministry of Education, and the volunteers were familiar with the protocol and procedure of the trial and obtained informed consent before the trial. The whole experiment was reviewed by the Sub-committee of Human Medical Experimentation Ethics of Guizhou University and ethical review notice was obtained. Volunteers exercised no more than 2 times per week in the 3 months before the test and did not perform strenuous exercise within 48 h before the test. The volunteers shaved the body hair around the RF and TA muscles of the lower limbs before the test, and wiped the skin at the sensor with 75% alcohol to reduce the low frequency noise caused by the movement of the sEMG collection electrodes due to sweat. The test volunteers and sampling equipment information are shown in Table 1 and Table 2.

#### 2.1.3. Experimental Design

**Figure 2:**
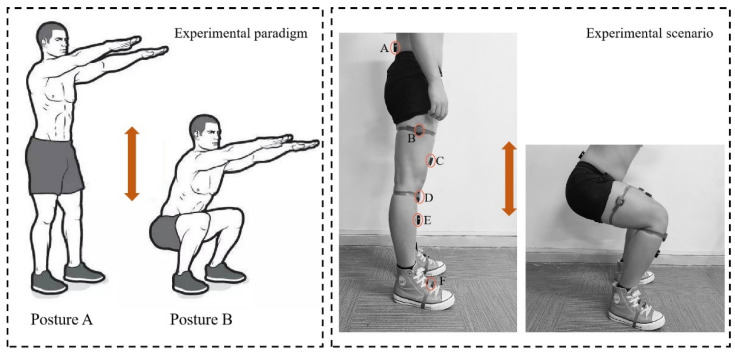
Fixed-length weightless lower-body squat exercise test.

In this paper, we designed a fixed-length unweighted squat exercise test with the test paradigm shown in Figure 2. Stance A is a standing stance with hands held forward, and Stance B is a squatting stance with the direction of the arrow as the direction of movement. Each subject performed a weightless squat test according to the test paradigm, defining a complete weightless squat from Posture A to Posture B and back to Posture A. Each subject performed two sets of tests, the first set lasting 30 s. After the first set of tests, the subject remained standing and adjusted breathing for 30 s, and then performed the second set of tests, the second set lasting 60 s. The speed of the squatting movement and the number of completed squatting movements were not fixed. The relevant signal acquisition sensors were placed as shown in Figure 2, where A, B, D and F are lower limb motion capture inertial sensors, and C and E are sEMG signal sensors. During the test, the hip joint angle information was acquired by sensors A and B, the knee joint angle information was acquired by sensors B and D, the ankle joint angle information was acquired by sensors D and F, the rectus femoris sEMG signal was acquired by sensor C, and the tibialis anterior sEMG signal was acquired by sensor F. The orange two-way arrow means the direction of the movement.

### 2.2. Methods

#### 2.2.1. Comfort Feature Index Extraction and Calculation

1. Psychological comfort index extraction

In this paper, we set up a 7-point Likert scale of subjective comfort to measure the subjective comfort of subjects, and obtain the ratings of the nth subject on the four questions in the scale at time *t*, according to the four questions set in the scale $Q1_{n}^{t}$, $Q2_{n}^{t}$, $Q3_{n}^{t}$, $Q4_{n}^{t}$, defined $Li_{n}^{t}=\{Q1_{n}^{t},Q2_{n}^{t},Q3_{n}^{t},Q4_{n}^{t}\}$ as the *n*th subjective perception Likert 7 scale score of the subject’s comfort at moment *t*. Principal component analysis [25] was performed on $Li_{n}^{t}$ using the factor analysis module of SPSS numerical analysis software (IBM Inc., Armonk, NY, USA), and the results are shown in Table 3.

As can be seen from Table 3, the percentage of variance of *Q*4 eigenvalues is the largest among the four questions set in the subjective comfort level Likert 7 scale, reaching 90.53%, *Q*1 is the second largest, reaching 7.13%, and the percentage of variance of *Q*2 eigenvalues is the smallest, at 0.75%. The results of the principal component analysis of the psychological comfort index were used to obtain the calculation of the subjective score of comprehensive psychological comfort, as shown in Equation (1).

(1) $$M_{n}^{t}=90.53\%*Q4_{n}^{t}+7.13\%*Q1_{n}^{t}+1.59\%*Q3_{n}^{t}+0.75\%*Q2_{n}^{t}$$

According to Equation (1), the subjective score $M_{n}^{t}$ of the combined psychological comfort of the *n*th subject at moment *t* was calculated, and Table 4 shows the $M_{n}^{t}$ and $Li_{n}^{t}$ of some subjects.

2. Skeletal muscle comfort index extraction

There is a high correlation between skeletal muscle fatigue and comfort perception [26], and muscle tone is the basis for maintaining different body postures and normal movements: the higher the muscle tone, the higher the fatigue and the lower the comfort level of the subject [27]. The theory of the EMG_FT_ algorithm, first proposed by Matsumoto et al. [9], refers to the use of sEMG and its analysis techniques to determine the motor muscle fatigue threshold. The results obtained by performing calculations on EMG_FT_ can be used to determine the anaerobic threshold (AT) [28] and the corresponding skeletal muscle loading intensity.

However, the traditional EMG_FT_ algorithm can only calculate the fatigue threshold time points of the corresponding skeletal muscles, and cannot quantitatively evaluate and analyze the fatigue degree at any time point in the whole process of limb movement. To address the above problems, this paper improves the traditional EMG_FT_ algorithm by equidistant sampling analysis in the time dimension, and quantitatively evaluates the fatigue degree at any time point in the whole process of limb movement by calculating the difference between the fitted straight lines.

Firstly, in this study, in order to reduce the influence of the original sEMG signal noise on the experimental results, the original sEMG signal is successively filtered out from high frequency noise as well as industrial frequency and harmonic interference by designing 0~500 HZ low-pass filter [29] and 49.5~50 Hz adaptive notch filter [30], and finally wavelet threshold denoising is performed, and the number of wavelet threshold denoising decomposition is chosen as the 4th layer [31]. The original sEMG signal collected in the experiment is pre-processed by the above method to establish the sEMG data set. Then, the time window, *tim*, and moving window, *mov*, are set, and the *RMS* value of sEMG is calculated by adding windows according to Equation (2). Then, the first-order least squares fitting method was used to fit the *RMS* data set for multiple groups, and two fitted straight lines were obtained for each group, and the group with the largest slope product of the two fitted straight lines in each group was screened, and the time node corresponding to its intersection point was the EMG_FT_. Finally, the difference between the two straight lines was calculated using the equidistant sampling analysis in the time dimension, which was the skeletal muscle fatigue score value at that time point.

(2) $$RMS=\sqrt{\frac{1}{N_{t}}\sum_{i=1}^{N_{t}}X_{i}^{2}}$$

In the improved EMG_FT_ fatigue level determination algorithm in this paper, let the original sEMG signal be *X* = {*x*1*, x*2,..., *xn*}, set the time window *tim* as 2 s, the moving window *mov* as 0.5 times *tim*, be the skeletal muscle fatigue score of the *n*th subject at time *t*, and $EL_{n}^{t}(R)$ be the right lower limb rectus femoris fatigue score, and $EL_{n}^{t}(A)$ be the right lower limb tibialis anterior fatigue score. $EL_{n}^{t}(A)$ is the fatigue score of the right lower limb anterior tibialis muscle, and the improved EMG_FT_ fatigue level determination algorithm is implemented as follows (Algorithm 1):
**Algorithm1:** Improved EMG_FT_ fatigue level determination algorithm**Input**: *X*, *tim*, *mov*, sampling frequency *f* **Output:** EMG_FT_, $EL_{n}^{t}$ **1. //Step1:sEMG Pre-processing** *X_LPF_* ← Low-pass filter (*X*), *f_LPF_* ∈ [0, 500 HZ]. *X_ANF_* ← Adaptive wave trap (*X_LPF_*), *f_ANF_* ∈ [49.5, 50 HZ]. *X_WT_* ← Wavelet Threshold Denoising (*X_ANF_*), Times of decomposition = 4. **2. //Step2: Build *RMS* dataset** *RMS* ← sqrt($\sum_{1}^{tim*f}X_{WT}^{2}$/tim*f).
**3. //Step3: Group Fitting *RMS* and Calculation EMG_FT_** Get the length of *RMS m.*
**for** *i* ∈ (1, *m* − 5).
     *Xi* ← *RMS* (1), …, *RMS* (*i*+4). 
     *Yi* ← *RMS* (*i*+5), …, *RMS* (end). 
     were fitted by first order least squares *Xi* and *Yi*, respectively, get *L_1i_*, *L_2i_*. 
     *K* = *kL_1i_* × *k L_2i_* The time node corresponding to the intersection of the line fitted to the set of data with the largest slope product *K* is calculated as EMG_FT_. The straight lines are *L_1max_*, *L_2max_* **end**
**4. //Step4: Calculation of skeletal muscle fatigue scores by isometric sampling analysis** 
  *s* is the number of samples 
  *l* ← *m*/*s.*
**for** *t* ∈ (1, 60) $EL_{n}^{t}$ ← *L_1max_* (*t* × *l*) − *L_2max_* (*t × l*) **end**

**Figure 3:**
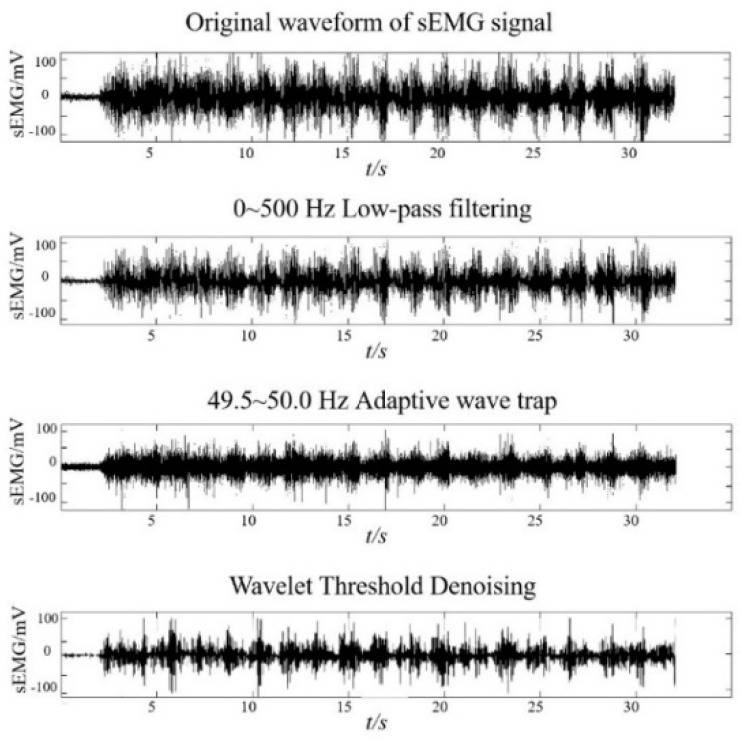
Time domain diagram of sEMG signal pre-processing process.

**Figure 4:**
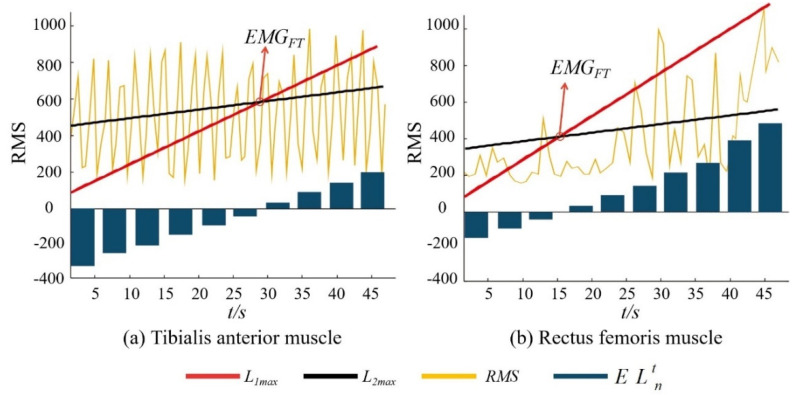
The result of improved EMG_FT_ fatigue level determination algorithm.

Figure 3 shows the time domain diagram of the sEMG signal preprocessing process, and Figure 4 shows the results of the improved EMG_FT_ fatigue rating algorithm for the same group of subjects in the fixed-length unweighted lower extremity squat test with setting *s* = 10.

As can be seen from Figure 4, the *RMS* values of the subjects during exercise showed an increasing trend with the onset of fatigue, the EMG_FT_ of RF appeared before the EMG_FT_ of TA, the fatigue time of TA was 29.6 s, and the fatigue time of RF was 15.3 s. The $EL_{n}^{t}$ of each time point is shown in the bar graph in the figure.

3. Joint comfort index extraction

The stability of the hip, knee, and ankle joints during fixed-length unweighted lower-body squats was correlated with the fatigue and comfort level of the subjects. The longer the exercise time, the lower the joint stability, the higher the fatigue, and the lower the comfort level [32]. In order to evaluate the comfort level of lower limb joints, this paper calculates joint stability by establishing a ball-and-stick model of weightless lower limb squatting exercise and defining the relevant joint angles based on the joint angle information. Using the joint angle information collected in the above experiments, the angular acceleration and its variance during the motion of each joint were calculated to evaluate the joint stability of each joint at any time point in the motion process.

**Figure 5:**
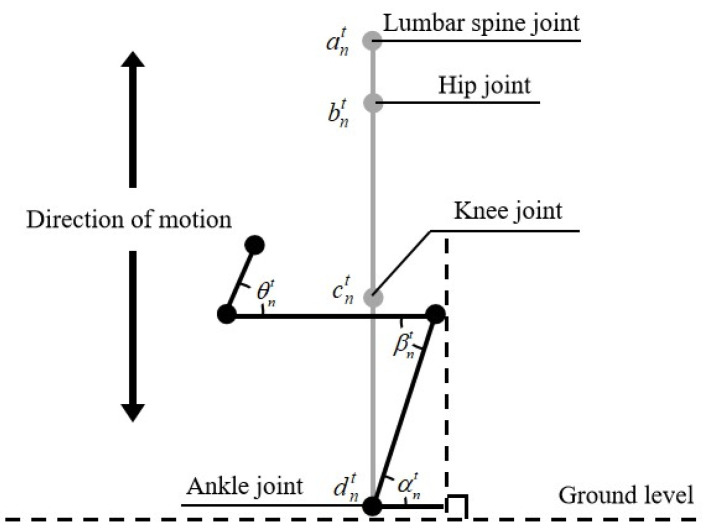
The sagittal lower limb squatting bat model.

In this paper, a ball-and-stick model with multiple rigid segments and hinge joints is used to establish a weightless lower limb squatting ball-and-stick model [33], and the angles of the joints of the lower limb are defined in the ball-and-stick model. The sagittal lower limb squatting bat model is shown in Figure 5.

As shown in the figure, $a_{n}^{t}$ is the lumbar joint point of the *n*th subject at time *t*, $b_{n}^{t}$ is the right lower limb hip joint point of the *n*th subject at time *t*, $c_{n}^{t}$ is the right lower limb knee joint point of the *n*th subject at time *t*, and $d_{n}^{t}$ is the right lower limb ankle joint point of the *n*th subject at time *t*. The hip joint angle, knee joint angle and ankle joint angle during the unweighted squatting exercise were defined according to the frontal plane unweighted squatting exercise ball and stick model and the spatial position of each joint point. Define the angle $\theta_{n}^{t}$ between vector $\vec{b_{n}^{t}a_{n}^{t}}$ and $\vec{b_{n}^{t}c_{n}^{t}}$ as the hip angle of the *n*th subject at the point of time *t*. Define the angle $\beta_{n}^{t}$ between vector $\vec{c_{n}^{t}b_{n}^{t}}$ and $\vec{c_{n}^{t}d_{n}^{t}}$ as the knee angle of the *n*th subject at the point of time *t*. Define the angle $\alpha_{n}^{t}$ between vector $\vec{d_{n}^{t}c_{n}^{t}}$ and the line where the ground plane is located as the ankle angle of the *n*th subject at the point of time *t*.

In order to evaluate the comfort level of hip, knee and ankle joints during weightless squatting, the angular acceleration and variance of each joint were calculated to reflect the stability of each joint during the weightless squatting exercise. Therefore, in this paper, the first-order least squares method [34] was used to fit the angular acceleration variance, and the joint comfort during weightless lower limb squatting was quantified at any time point by equidistant sampling analysis in the time dimension of the fitted line, with *s* being the number of sampling times, and the calculated hip joint comfort $D(H_{n}^{t})$, knee joint comfort $D(K_{n}^{t})$ and ankle joint comfort $D(A_{n}^{t})$ were used as the lower limb joint angular acceleration variance comfort index. Taking $D(H_{n}^{t})$ as an example, the specific algorithm is implemented as follows (Algorithm 2).
**Algorithm 2:** Lower extremity joint angular velocity variance comfort index algorithm**Input** $\theta_{n}^{t}$, $\beta_{n}^{t}$, $\alpha_{n}^{t}$, *tim*, *mov*, Sample frequency *f*, *s* **Output:**$D(H_{n}^{t})$
1. //Step1: Angular velocity calculation
$V(H_{n}^{t})$ ← $\Delta \theta_{n}^{t}/tim$
2. //Step2: Calculation of angular acceleration
$a(H_{n}^{t})$ ← $\Delta (V(H_{n}^{t}))/tim$
3. //Step3: Calculation of angular acceleration variance
Get the length of $a(H_{n}^{t})$
*m*
k = f*tim
*l* ← *m*/*k*
**for**
*i* ∈ (1,*k*) 
     variance $\left(H_{n}^{t}\right)$ ← $\frac{\sum_{i=1}^{i*k}\left(a{\left(H_{n}^{t}\right)}_{i}-mean(a{\left(H_{n}^{t}\right)}_{i}:a{\left(H_{n}^{t}\right)}_{i*k}\right)}{l}$ 
**end**

4. //Step4: Joint comfort calculation
Fitting by first-order least squares variance $\left(H_{n}^{t}\right)$, get *L_H_*
*t* = *m/s*
**for**
*i* ∈ (1,*s*) 
     $V(H_{n}^{t})$ ← F(*L_Hi_*: L_Hi*s_)
**end**

**Figure 6:**
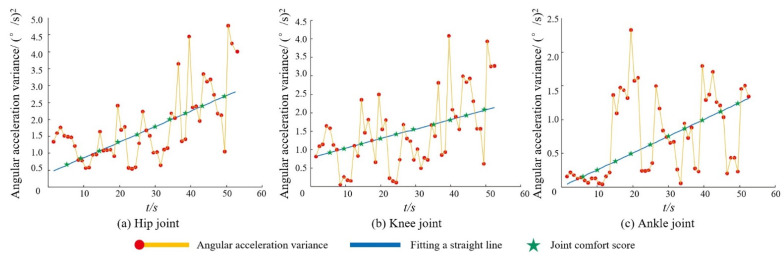
Results of the lower limb joint angular velocity variance comfort index algorithm.

Figure 6 shows the output of the lower extremity joint angular acceleration variance comfort index algorithm for a fixed duration unweighted lower extremity squat test with s = 10 for one of the randomized subjects.

As can be seen from Figure 6, the variance of each joint shows a general upward trend during movement. The straight line obtained by fitting the variance of each joint represents the trend of the variance of that joint, and the comfort score of each joint can be obtained by equidistant sampling on the time dimension of the resulting fitted straight line.

4. Lower extremity comfort level determination dataset construction

Firstly, the psychological comfort as well as physical comfort feature indicators $M_{n}^{t}$, $EL_{n}^{t}(R)$, $EL_{n}^{t}(A)$, $D(H_{n}^{t})$, $D(K_{n}^{t})$, $D(A_{n}^{t})$, extracted and calculated in 1 to 3 in Section 2.2.1, were sorted according to the time dimension. In addition, due to the lack of comparability and uniformity of the numerical intervals in which the original various types of comfort feature indicators were located, this paper applies Equation (3) to mean normalization [35] for each type of data, so that the different dimensions between the features are mapped to values between −1 and 1, and the comfort features data set are retained to construct the lower limb comfort features data set. By collating the obtained data, a group of comfort feature indexes and part of the lower limb comfort feature data set is shown in Table 5.

(3) $$X^{\prime }=\frac{X-mean(X)}{Max(X)-Min(X)}$$

In Equation (3), *X* is the comfort characteristic index, *X′* is the result of min-max normalization of the comfort characteristic index, *mean(X)* is the average value of the class of comfort characteristic index, *Max(X)* is the maximum value of the class of comfort characteristic index, and *Min(X)* is the minimum value of the class of comfort characteristic index.

Then, in order to determine the comfort level of lower limb movement from two dimensions, psychological comfort and physiological comfort, a lower limb movement comfort classification model needs to be established through supervised learning, and before establishing the comfort level determination model, label values need to be constructed. The higher the score of psychological comfort, the lower the comfort level, and the higher the score of joint stability, the lower the comfort level, and each feature value is at (−1,1) after normalization, so the lower limb comfort feature values are clustered by clustering method to construct labels. In order to better classify the comfort status, the results are clustered into 4 classes in this paper. The relationship of the comfort degree and comfort level is shown in Table 6.

K-means clustering algorithm is a common traditional clustering algorithm, but due to the excessive computational complexity of too many iterations [36], it will lead to slow convergence, in order to improve the convergence effect and reliability of the clustering model, this paper uses the K-means++ algorithm [37] to produce the initial clustering centers generated by the algorithm away from each other, optimize the initial clustering centers, resulting in a more reliable algorithm than the traditional K-means clustering algorithm The clustering results are more reliable than the traditional K-means clustering algorithm.

Since the lower limb comfort features dataset is a 6-dimensional dataset with high dimensionality, it will have an impact on the effective training efficiency of the lower limb motion comfort classification model, so this paper deconstructs the lower limb comfort features dataset into psychological comfort features and physiological comfort features through hierarchical analysis, the psychological comfort features include the comprehensive psychological comfort subjective scores, the physiological comfort features include The mental comfort features include the comprehensive psychological comfort subjective score, the physical comfort features include the skeletal muscle comfort features and the joint comfort features, and the principal component analysis was performed on these three types of features, and the results of the comfort features classification and principal component analysis are shown in Table 7, and then one feature was selected in each type of features according to the results of the principal component analysis to form a 3-dimensional lower limb comfort features dataset, and the extracted comfort features were $M_{n}^{t}$, $EL_{n}^{t}(R)$, $D(K_{n}^{t})$ according to the percentage of variance of the feature values shown in Table 7. Finally, the reduced-dimensional lower limb comfort features dataset was subjected to K-means++ clustering, and *k* = 4 was set in the K-means++ clustering algorithm.

To demonstrate that dimensionality reduction in the eigenvalues using principal component analysis does not degrade the performance of the K-means++ algorithm for clustering, this study clustered the original six eigenvalues as well and compared the clustering results with the clustering results of the eigenvalues after dimensionality reduction, and the overlap rates of the clustering results are shown in Table 8. From Table 8, it can be seen that the overlap rate of the clustering results before and after the dimensionality reduction is high, so the dimensionality reduction in the eigenvalues does not reduce the performance of the clustering algorithm.

**Figure 7:**
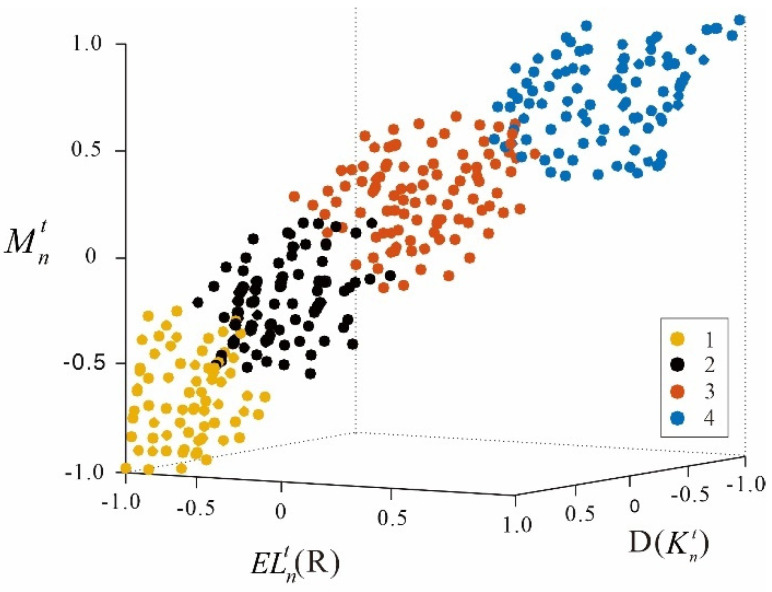
K-means++ clustering result.

Define the K-means++ clustering results as $KK_{n}^{t}$, and the clustering results are shown in Figure 7. Levels 1, 2, 3 and 4 in the clustering results correspond to 4 comfort scores, and the higher the score, the lower the comfort level. The lower limb comfort level determination dataset is jointly constructed by merging with the reduced-dimensional lower limb comfort feature dataset.

#### 2.2.2. QUEST Algorithm and Its Principle

Decision tree is a supervised machine learning classification algorithm that represents the classification logic of things by forming a tree diagram through a recursive algorithm [38]. QUEST (Quick, Unbiased, Efficient Statistical Tree) is a fast, unbiased and efficient statistical decision tree, a binary tree algorithm. It uses ANOVA F [39] or Pearson’s chi-square test [40] to select split variables [41].

In this part, $M_{n}^{t}$, $EL_{n}^{t}(R)$, $D(K_{n}^{t})$ in the comfort level determination dataset constructed in Section 3.1 are used as independent variables and $KK_{n}^{t}$ as dependent variables to establish a QUEST-based upper limb motion comfort level analysis and determination model, as follows [42].

1. So that the significance level α∈(0,1), *M* is the number of variables and *M*1 is the number of continuous and ordered variables.
2. By performing ANOVA F-test on all continuous or ordered independent variables *X*, it was tested whether the dependent variables of different categories had the same mean value as *X* and the smallest *p*-value was screened according to the Pearson chi-square statistic.
3. Pearson chi-square tests for independence of *Y* and *X* were performed for each category of independent variables, and *p*-values were derived from the Pearson chi-square statistic.
4. The independent variable with the smallest *p*-value is filtered out, and this independent variable is denoted by X*.
5. If this minimum *p*-value is less than, select the independent variable X* as the predictor of the split node. If not, perform 3.
6. For each continuous independent variable *X*, Levene’s test [43] is performed to detect whether the variance of *X* is the same for different types of *Y* based on the absolute deviation of *X* from its class mean, and the *p*-value is found.
7. The independent variable with the smallest *p*-value is filtered out, and this independent variable is denoted by X**.
8. If this minimum *p*-value is less than, then X** is selected as the splitting independent variable of the node, otherwise, the node is not split.

The equation for the Pearson chi-square statistic is shown in Equation (4).

(4) $$x^{2}=\sum_{i,j}\frac{{\left(O_{i,j}-E_{i,j}\right)}^{2}}{E_{\bar{i,j}}}$$

In Equation (4), *O_i,j_* is the observed value, and *E_i,j_* is the expected value of row *i* and column *j*.

The equation for the ANOVA F-statistic is shown in Equation (5).

(5) $$F=\frac{\sum nj{\left(\bar{X}j-\bar{X}\right)}^{2}/(k-1)}{\sum \sum {\left(X-\bar{X}j\right)}^{2}/(N-k)}$$

In Equation (5), *nj* is the sample size of the *j*th group, ${\bar{X}}_{j}$ is the sample mean, $\bar{X}$ is the overall mean, *N* is the total number of observations, and *k* is the number of independent groups.

## 3. Results

### 3.1. QUEST-Based Upper Limb Motion Comfort Level Analysis and Determination Model and Results

In this part, in order to conduct a comprehensive level analysis and determination of lower limb exercise comfort from two dimensions, psychological comfort and physiological comfort, a QUEST-based upper limb exercise comfort level analysis and determination model needs to be established based on the lower limb comfort level determination dataset constructed in Section 2.2.1 of this paper. Information on the QUEST-related algorithm is shown in Table 9.

**Figure 8:**
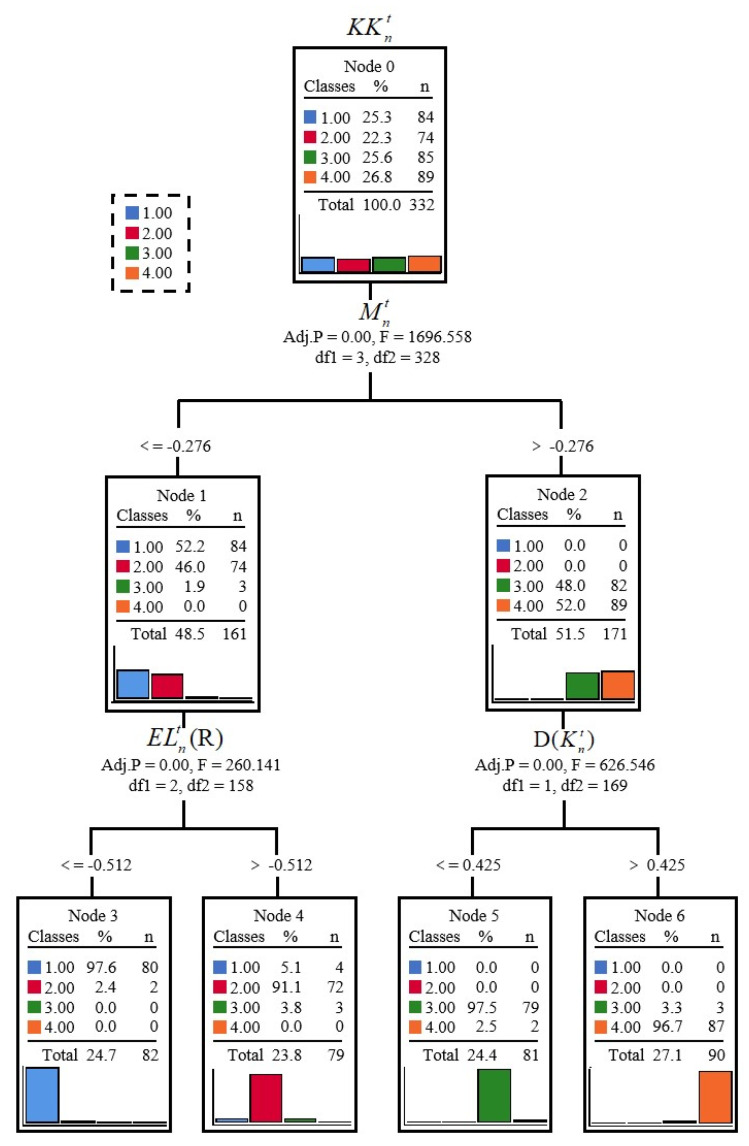
QUEST-based lower limb motion comfort level analysis and determination model.

Through the QUEST decision tree module of IBM SPSS Statistics 26 numerical analysis software (Armonk, NY, USA), the QUEST-based upper limb motion comfort level analysis and determination model was established as shown in Figure 8, the model prediction accuracy is shown in Table 10, and the risk values are shown in Table 11.

As shown in Figure 8, the QUEST-based upper limb motion comfort level analysis and determination model ended with four endpoints, seven nodes, and a depth of two. As shown in Table 7, the model achieved a comprehensive accuracy of 95.8% for the classification of lower limb motion comfort level, where the classification accuracy for comfort score 1.00 was 95.2%; the classification accuracy for comfort score 2.00 reached 97.3%; the classification accuracy for comfort score 3.00 was lower, reaching 92.9%; the classification accuracy for comfort score 4.00 was the highest, reaching 97.8%. As shown in Table 8, the prediction accuracy value (risk value) of the model was 0.042, which means that a 4.2% misclassification occurred overall.

The following conclusions can be summarized by analyzing the nodes in Figure 8 as well as the split.

Node 0: Node 0 is the dependent variable $KK_{n}^{t}$. There are 332 lower extremity motor comfort level scores in $KK_{n}^{t}$, of which 84 are 1.00, accounting for 25.3% of the total; 74 are 2.00, accounting for 22.3% of the total; 85 are 3.00, accounting for 25.6% of the total; and 89 are 4.00, accounting for 26.8% of the total. The proportion of each type of score is more balanced.

Node 1: Node 1 is obtained by splitting the independent variable $M_{n}^{t}$, which indicates the part of $M_{n}^{t}$ eigenvalues with values less than or equal to 0.276. In this node, there are: 84 scores of 1.00, accounting for 52.2% of the total in this node; 74 scores of 2.00, accounting for 46.0% of the total in this node; 3 scores of 3.00, accounting for 1.9% of the total in this node. Therefore, this node is considered as a node with a classification of 1.00 points as well as 2.00 points.

Node 2: Node 2 is obtained by splitting the independent variable $M_{n}^{t}$ and represents the fraction of $M_{n}^{t}$ eigenvalues with values greater than 0.276. In this node, there are 82 scores of 3.00, accounting for 48.0% of the total in this node, and 89 scores of 4.00, accounting for 52.0% of the total in this node. Therefore, this node is used as a node with a classification of 3.00 points as well as 4.00 points.

Node 3: Node 3 is obtained by splitting node 1 as well as the independent variable $EL_{n}^{t}(R)$. It represents the portion of $EL_{n}^{t}(R)$ eigenvalues with values less than or equal to −0.521. In this node, there are 80 scores of 1.00, accounting for 97.6% of the total in this node, and 2 scores of 2.00, accounting for 2.40% of the total in this node, with an overall percentage of 24.7%. Therefore, this node is used as the node with a classification of 1.00 points.

Node 4: Node 4 is obtained by splitting node 1 as well as the independent variable $EL_{n}^{t}(R)$. It represents the fraction of $EL_{n}^{t}(R)$ eigenvalues with values greater than −0.521. In this node, there are 4 scores of 1.00, accounting for 5.1% of the total in this node, and 72 scores of 2.00, accounting for 91.1% of the total in this node, with an overall percentage of 23.8%. Therefore, this node was used as the node with a classification of 2.00 points.

Node 5: Node 5 is obtained by splitting node 2 as well as the independent variable $D(K_{n}^{t})$. It represents the portion of $D(K_{n}^{t})$ eigenvalues with values less than or equal to 0.425. In this node, there are 79 scores of 3.00, accounting for 97.5% of the total in this node, and 2 scores of 4.00, accounting for 2.5% of the total in this node, with an overall percentage of 24.4%. Therefore, this node was used as the node with a classification of 3.00 points.

Node 6: Node 6 is obtained by splitting node 2 as well as the independent variable $D(K_{n}^{t})$. It represents the fraction of $D(K_{n}^{t})$ eigenvalues with values greater than 0.425. In this node, there are 3 scores of 3.00, accounting for 3.3% of the total in this node, and 87 scores of 4.00, accounting for 96.7% of the total in this node, with an overall percentage of 27.1%. Therefore, this node is used as the node with a classification score of 4.00.

By analyzing the model classification results, we can learn that the comfort subjective score $M_{n}^{t}$ is able to classify the comfort levels as 1 and 2 and 3 and 4, but it is not able to carry out the four levels of classification basis, indicating that the comfort subjective score does not provide a complete classification of the comfort levels. The skeletal muscle comfort score $EL_{n}^{t}(R)$ was able to classify comfort levels 1 and 2, and the joint comfort score $D(K_{n}^{t})$ was able to classify comfort levels 3 and 4, suggesting that skeletal muscle discomfort occurs earlier than joint discomfort during unweighted squats and is more sensitive to comfort scores 1 and 2. The results suggest that this paper is correct in considering the effects of subjective comfort perceptions, skeletal muscle comfort perceptions and joint comfort perceptions on comfort perceptions during lower limb exercise, and that a combination of psychological and physical comfort indicators is more effective and accurate than a single indicator.

### 3.2. Comparison with Other Classification Algorithms

**Figure 9:**
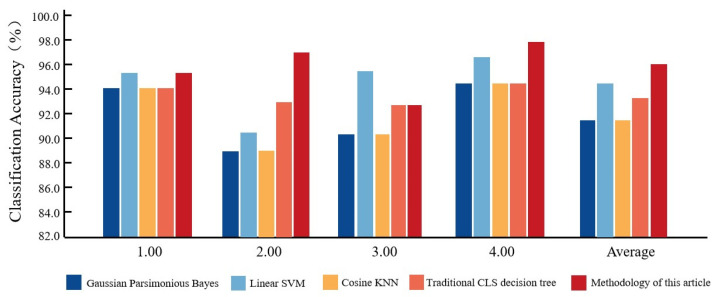
Each algorithm classification accuracy comparison of results.

To verify the advantages of the QUEST-based lower extremity motion comfort level analysis and determination model proposed in this paper in lower extremity comfort level analysis, four supervised classification algorithms, Gaussian plain Bayes [20], linear SVM [21], cosine KNN [22] and traditional CLS decision tree [23], were trained on the basis of the comfort level determination dataset constructed in Section 2.2.1, and the model results were compared with the results of the QUEST-based lower extremity motion comfort level analysis and determination model proposed in this paper. The results of QUEST-based lower limb motion comfort level analysis and determination model were compared. The relevant comparison algorithm information is shown in Table 12, the classification accuracy of each comparison algorithm for the four comfort scores is shown in Table 13, and the results of each comparison algorithm compared with the method in this paper are shown in Figure 9.

Based on the analysis of the training results of each comparative algorithm model, the following conclusions can be drawn.

1. In the classification of 1.00 comfort level, linear SVM and the method in this paper have the highest classification accuracy of 95.2%, and the classification accuracy of the remaining comparison algorithms is 94.0%. The results show that the classification effect of each comparison algorithm and the method in this paper is relatively average for 1.00 comfort level, among which linear SVM and the method in this paper have the best performance.
2. In the classification of 2.00 comfort level, the classification accuracy of this method is the highest, reaching 97.3%. Among the rest of the compared algorithms, the recognition rate of traditional CLS decision tree is higher, reaching 93.2%, and the recognition rate of Gaussian Parsimonious Bayes and cosine KNN is lower, only 87.85%.
3. In the classification of 3.00 comfort level, the linear SVM has the highest classification accuracy of 95.3%, the method of this paper and the cosine KNN classification accuracy of 92.9%, which is second only to the linear SVM, and the Gaussian plain Bayesian classification accuracy is lower, only 90.6%. The results show that the linear SVM has the best classification of 3.00 comfort level.
4. In the classification of 4.00 comfort level, the classification accuracy of the method in this paper is the highest, reaching 97.8%, and among the remaining comparison algorithms, the linear SVM classification accuracy is higher, reaching 96.6%, and the classification accuracy of the remaining comparison algorithms is 94.4%. The results show that the method in this paper has the best classification for the 4.00 comfort level.
5. From the summary classification accuracy, it can be seen that the method in this paper has the highest classification accuracy for comfort level, reaching 95.8%, which is higher than other comparison algorithms. Among other comparison algorithms, linear SVM has a higher recognition rate, reaching 94.4%, and the results show that the method in this paper has a better classification effect for lower limb exercise comfort level analysis and determination, and can achieve an effective level classification for lower limb exercise comfort.

## 4. Discussion

Lower limb movement is a kind of complex movement which has multi-dimensional and multi-degree freedom. It is necessary to introduce the comfort level as a reference index into many fields, such as designing and optimizing lower limb-assistive sports equipment and comfort estimation of lower limb rehabilitation medical treatment. However, due to the lack of comprehensive and effective quantitative evaluation standards for the comfort level in the process of lower limb movement, most of the relevant studies have evaluated the comfort level of the lower limb from only single psychological or physiological comfort index. The crux of the matter is human comfort feeling is not a single psychological or physiological response, but a multidimensional fusion of human feelings. Therefore, in order to resolve the fuzzy and one-sidedness problem of the comfort judgment, this paper obtained and analyzed the psychological comfort and physiological comfort indexes to establish a lower limb movement comfort degree data set. Finally, we establish a decision tree model for lower limb comfort level analysis and determination, which incorporates both psychological comfort indexes and physiological comfort indexes into the consideration of lower limb comfort level judgment. The following points were found in the study.

1. In this paper, the EMG_FT_ algorithm was used in the process of extracting the feature values of skeletal muscle comfort, and for its inability to quantify the fatigue degree level at any time point in the time dimension, an improvement was proposed to realize the quantitative analysis of the fatigue degree at any time point during lower limb exercise, which provided data support for the later study and also provided a new idea for the EMG_FT_ algorithm to conduct the evaluation of skeletal muscle fatigue level.
2. Since a single skeletal muscle fatigue state cannot accurately reflect the comfort perception of lower limbs, this paper introduces lower limb joint stability monitoring and innovatively evaluates the stability of joints during motion by calculating the variance of angular acceleration of lower limb joints to achieve the comfort evaluation of lower limb joints.
3. In order to comprehensively consider the influence of psychological factors and physiological factors on the determination of lower limb comfort level, this paper selects $M_{n}^{t}$, $EL_{n}^{t}(R)$, $EL_{n}^{t}(A)$, $D(H_{n}^{t})$, $D(K_{n}^{t})$ and $D(A_{n}^{t})$ from the psychological comfort, skeletal muscle comfort and joint comfort, respectively, to carry out relevant research, and filters out $M_{n}^{t}$, $EL_{n}^{t}(R)$, $D(K_{n}^{t})$, three comfort characteristic indexes, by principal component analysis, which realizes the dimensionality reduction processing of the data set at the same time. It can be seen that the lower limb rectus femoris and lower limb knee joint have a greater influence on the lower limb movement state during the lower limb squatting movement.
4. In the QUEST-based lower limb exercise comfort level analysis and determination model, it can be seen that $M_{n}^{t}$ in the independent variable is the node that splits first, and the node is the basis for the judgment of comfort level 1.00 and 2.00 and comfort level 3.00 and 4.00, which indicates that the psychological comfort index can make a two-level judgment of the comfort level during lower limb exercise, but it does not provide a detailed comfort feeling grading. $EL_{n}^{t}(R)$ and $D(K_{n}^{t})$ are needed to produce a detailed classification of comfort level, and the skeletal muscle comfort index $EL_{n}^{t}(R)$ has higher contribution to the classification of comfort level 1.00 and 2.00, and the joint comfort index $D(K_{n}^{t})$ has higher contribution to the classification of comfort level 2.00 and 3.00, indicating that the single psychological comfort evaluation is more ambiguous during lower limb movement, and the skeletal muscle discomfort feelings appear earlier than the uncomfortable feelings of the joints.
5. In the comparison with other supervised classification algorithms, it is found that the method of this paper has obvious advantages, especially in the recognition and classification of comfort level 2.00 and 4.00. By comparing the classification accuracy of this paper and the comparison algorithms for each comfort level, it can be found that all types of algorithms are more stable and more accurate for the recognition of comfort level 1.00 and 4.00, and the classification accuracy is higher for the comfort level 2.00 and 3.00. For the transitional comfort level 2.00 and 3.00, the classification rate is lower. It means that the comfort feature values obtained from the data set of this paper are more accurate for the evaluation of comfortable and uncomfortable states, but more vague for the evaluation of transitional states.

However, there are several shortcomings in the methodology of this paper as follows:

1. In this paper, a fixed-length weightless lower limb squat test was used as the test paradigm to obtain the comfort characteristics data set, and the test subjects’ age and gender were relatively limited. However, there are many kinds of lower limb movements, and a single lower limb squatting movement does not reflect the complete lower limb movement status. In the next study, all kinds of lower limb movements should be used as the test paradigm, and the effects of different types of test subjects should be considered to increase the reliability of the dataset.
2. The unstable clustering results of the K-means++ algorithm leads to a more ambiguous classification of the transition comfort state in the lower limb comfort level determination dataset, and in the next study, reference variables for classifying the transition state should be introduced to improve the reliability of the lower limb comfort level determination dataset.
3. Although the method in this paper can achieve the classification of comfort states during lower limb squatting exercise, the accuracy of the classification of transitional comfort states needs to be improved.

In summary, the QUEST-based lower limb exercise comfort level analysis and determination model proposed in this paper can realize the monitoring of comfort status and comfort level determination during lower limb squatting exercise and provide quantitative comfort indexes, which can provide theoretical support for comfort status monitoring in the fields of lower limb exercise analysis, related lower limb exercise aids and lower limb medical rehabilitation.

## 5. Conclusions

The purpose of this paper is to establish a QUEST decision tree model for analyzing and judging lower limb movements. In this model, the eigenvalues of physiological comfort and psychological comfort are simultaneously considered in this model to comprehensively judge the lower limbs comfort during exercise. The comprehensive classification accuracy of the model reaches 95.8%, of which the classification accuracy of the four comfort levels reaches 95.2%, 97.3%, 92.9%, and 97.8%, respectively, realizing the effective comfort level analysis during the lower limb squatting movement and judgment. In the following related research work, factors such as different lower extremity movement patterns and the age and gender of the subjects should be taken into consideration to improve the reliability of the comfort level judgment dataset. The reference variable for classifying transition comfort state is introduced to improve the classification accuracy of the model for the transition comfort state during lower extremity movement, and to better realize multi-dimensional monitoring and grade determination of lower extremity movement comfort state.

In summary, the QUEST-based lower limb exercise comfort level analysis and determination model proposed in this paper can achieve effective comfort level determination and classification in the process of lower limb squatting exercise, which puts forward a new idea for lower limb exercise comfort status monitoring and largely remedies the problem of ambiguity in the evaluation of comfort perception of a single comfort index, and also provides theoretical support for subsequent research.

## Figures and Tables

**Table 1:** Relevant subject information.

**Volunteers** **information**	**Quantity**	**Gender**	**Age**	**Height (cm)**	**Weight (kg)**	**BMI**
	25	Male	22.4 ± 1.5	173.2 ± 5.8	65.3 ± 7.2	22.7 ± 1.8

**Table 2:** Test equipment information.

**Sampling equipment information**	**Name**	**Sampling Data**	**Sampling Frequency (Hz)**	**Quantity**
	Delsys TrignoTM Wireless EMG	VR’s sEMGTA’s sEMG	1925	4
		Hip angleKnee AngleAnkle angle	75	
	Subjective scale of comfort	Subjective rating	0.1	4

**Table 3:** Results of principal component analysis of psychological comfort index.

**Subjective Feeling of Comfort**	$Li_{n}^{t}$			
	$Q1_{n}^{t}$	$Q2_{n}^{t}$	$Q3_{n}^{t}$	$Q4_{n}^{t}$
**Eigenvalue variance percentage**	7.13%	0.75%	1.59%	90.53%

**Table 4:** The $M_{n}^{t}$ and $Li_{n}^{t}$ of some subjects.

	$Q1_{n}^{t}$	$Q2_{n}^{t}$	$Q3_{n}^{t}$	$Q4_{n}^{t}$	$M_{n}^{t}$
01	3.2	4.6	4.7	4.2	4.14
02	6.2	5.8	5.7	6.3	6.28
03	2.1	2.6	3.0	2.8	2.75
04	5.1	5.1	5.6	5.4	5.38
05	4.7	3.9	4.7	5.0	4.97

**Table 5:** Partial lower extremity comfort feature dataset.

	$M_{n}^{t}$	$EL_{n}^{t}(R)$	$EL_{n}^{t}(A)$	$D(H_{n}^{t})$	$D(K_{n}^{t})$	$D(A_{n}^{t})$
**01**	0.20	0.49	0.20	0.53	0.45	0.49
**02**	−0.23	0.49	0.20	0.55	0.45	0.50
**03**	0.16	0.50	0.22	0.56	0.46	0.50
**04**	0.05	0.52	0.22	0.57	0.47	0.50
**05**	0.25	0.55	0.24	0.58	0.47	0.51
**06**	0.20	0.55	0.25	0.58	0.50	0.52
**07**	0.33	0.59	0.26	0.59	0.50	0.53
**08**	0.32	0.61	0.26	0.60	0.51	0.54
**09**	−0.07	0.62	0.27	0.60	0.51	0.54
**10**	0.19	0.64	0.28	0.60	0.51	0.54
**11**	−0.16	0.66	0.29	0.61	0.51	0.55
**12**	−0.28	0.66	0.29	0.61	0.53	0.55
**13**	0.22	0.67	0.33	0.63	0.54	0.55
**14**	0.05	0.67	0.33	0.63	0.55	0.56
**15**	0.04	0.67	0.33	0.64	0.56	0.56

**Table 6:** Partial lower extremity comfort feature dataset.

Comfort Degree	Comfortable	Mildly Uncomfortable	More Uncomfortable	Entire Uncomfortable
Comfort level	1	2	3	4

**Table 7:** Classification of comfort features and results of principal component analysis.

Category Classification	Psychological Comfort Characteristics	Physiological Comfort Characteristics				
		Skeletal Muscle Comfort Characteristics		Joint Comfort Characteristics		
**Eigenvalue**	$M_{n}^{t}$	$EL_{n}^{t}(R)$	$EL_{n}^{t}$ **(A)**	$D(H_{n}^{t}$)	$D(K_{n}^{t}$)	$D(A_{n}^{t}$)
**Eigenvalue variance percentage**	100	98.42	1.57	13.24	86.32	0.43

**Table 8:** Comparison of clustering results before and after dimensionality reduction.

Comfort Levels	Overlap before and after Dimensionality Reduction
1	98.4%
2	99.5%
3	98.7%
4	99.2%

**Table 9:** Information on QUEST-related algorithms.

Research Parameters	Information
**Data Analysis Model**	Logistic regression: decision tree and QUEST algorithm
**Test platform**	IBM SPSS Statistics 26
**Dependent variable**	$KK_{n}^{t}$
**Independent variable**	$M_{n}^{t}$ $;\;EL_{n}^{t}(R)$ $;\;D(K_{n}^{t})$
**Verification method**	Cross-validation
**Splitting Rules**	ANOVA F-statistic

**Table 10:** Prediction accuracy of QUEST-based lower extremity motion comfort level analysis and determination model.

Real Test	Projections				Percent Correct
	1.00	2.00	3.00	4.00	
**1.00**	80	4	0	0	95.2%
**2.00**	2	72	0	0	97.3%
**3.00**	0	3	79	3	92.9%
**4.00**	0	0	2	87	97.8%
**Overall percentage**	24.7%	23.8%	24.4%	27.1%	95.8%

**Table 11:** Prediction accuracy values of QUEST-based lower limb motion comfort level analysis and determination model.

Method	Estimate	Standard Error
Re-substitute	0.042	0.011
Cross-validation	0.072	0.014

**Table 12:** Information on relevant comparison algorithms.

Algorithm Information	Comparison Algorithm			
	Gaussian Parsimonious Bayes	Linear SVM	Cosine KNN	Traditional CLS Decision Tree
**Tags**	$KK_{n}^{t}$	$KK_{n}^{t}$	$KK_{n}^{t}$	$KK_{n}^{t}$
**Predictive variables**	$M_{n}^{t}$ $;\;EL_{n}^{t}(R)$ $;\;D(K_{n}^{t})$	$M_{n}^{t}$ $;\;EL_{n}^{t}(R)$ $;\;D(K_{n}^{t})$	$M_{n}^{t}$ $;\;EL_{n}^{t}(R)$ $;\;D(K_{n}^{t})$	$M_{n}^{t}$ $;\;EL_{n}^{t}(R)$ $;\;D(K_{n}^{t})$
**Verification method**	Cross-checking	Cross-checking	Cross-checking	Cross-checking
**Classification basis**	posterior probability	Linear kernel functions	Distance Metric	Information Gain

**Table 13:** Classification accuracy of each comparison algorithm.

Comfort Level	Comparison Algorithm			
	Gaussian Parsimonious Bayes	Linear SVM	Cosine KNN	Traditional CLS Decision Tree
**1.00**	94.0%	95.2%	94.0%	94.0%
**2.00**	87.8%	90.5%	87.8%	93.2%
**3.00**	90.6%	95.3%	90.6%	92.9%
**4.00**	94.4%	96.6%	94.4%	94.4%
Average	91.7%	94.4%	91.7%	93.6%

## Data Availability

Data are available on request, due to privacy and ethical restrictions. Main data is contained within the article.
